# mTORC1 and mTORC2 Differentially Regulate Cell Fate Programs to Coordinate Osteoblastic Differentiation in Mesenchymal Stromal Cells

**DOI:** 10.1038/s41598-019-56237-w

**Published:** 2019-12-27

**Authors:** Theres Schaub, Dennis Gürgen, Deborah Maus, Claudia Lange, Victor Tarabykin, Duska Dragun, Björn Hegner

**Affiliations:** 1 Clinic for Nephrology and Intensive Care Medicine, Charité-Universitätsmedizin Berlin, corporate member of Freie Universität Berlin, Humboldt-Universität zu Berlin, and Berlin Institute of Health, Berlin, Germany; 2 Institute for Chemistry and Biochemistry, Freie Universität Berlin, Berlin, Germany; 3 Institute of Cell Biology and Neurobiology, Charité-Universitätsmedizin Berlin, corporate member of Freie Universität Berlin, Humboldt-Universität zu Berlin, and Berlin Institute of Health, Berlin, Germany; 4 Center for Cardiovascular Research (CCR), Charité University Hospital, Berlin, Germany; 5 Experimental Pharmacology & Oncology Berlin-Buch GmbH, Berlin, Germany; 6 Junior Research Group 2: Metabolism of Microbial Pathogens, Robert Koch Institute, Berlin, Germany; 7 Clinic for Stem Cell Transplantation, Department of Cell and Gene Therapy, University Medical Center Hamburg-Eppendorf, Hamburg, Germany; 8 Berlin-Brandenburg School for Regenerative Therapies (BSRT), Berlin, Germany; 9 Vivantes Ida Wolff Hospital for Geriatric Medicine, Berlin, Germany

**Keywords:** Cell signalling, Calcification, Cardiovascular diseases, End-stage renal disease

## Abstract

Vascular regeneration depends on intact function of progenitors of vascular smooth muscle cells such as pericytes and their circulating counterparts, mesenchymal stromal cells (MSC). Deregulated MSC differentiation and maladaptive cell fate programs associated with age and metabolic diseases may exacerbate arteriosclerosis due to excessive transformation to osteoblast-like calcifying cells. Targeting mTOR, a central controller of differentiation and cell fates, could offer novel therapeutic perspectives. In a cell culture model for osteoblastic differentiation of pluripotent human MSC we found distinct roles for mTORC1 and mTORC2 in the regulation of differentiation towards calcifying osteoblasts via cell fate programs in a temporally-controlled sequence. Activation of mTORC1 with induction of cellular senescence and apoptosis were hallmarks of transition to a calcifying phenotype. Inhibition of mTORC1 with Rapamycin elicited reciprocal activation of mTORC2, enhanced autophagy and recruited anti-apoptotic signals, conferring protection from calcification. Pharmacologic and genetic negative interference with mTORC2 function or autophagy both abolished regenerative programs but induced cellular senescence, apoptosis, and calcification. Overexpression of the mTORC2 constituent rictor revealed that enhanced mTORC2 signaling without altered mTORC1 function was sufficient to inhibit calcification. Studies in mice reproduced the *in vitro* effects of mTOR modulation with Rapamycin on cell fates in vascular cells *in vivo*. Amplification of mTORC2 signaling promotes protective cell fates including autophagy to counteract osteoblast differentiation and calcification of MSC, representing a novel mTORC2 function. Regenerative approaches aimed at modulating mTOR network activation patterns hold promise for delaying age-related vascular diseases and treatment of accelerated arteriosclerosis in chronic metabolic conditions.

## Introduction

Vascular regeneration depends on local vascular smooth muscle cell (VSMC) precursor cells, or pericytes^1–3^, that are replenished by mesenchymal stromal cells (MSC)^4^. MSC are progenitor cells featuring multi-lineage differentiation potential including VSMC^5,6^ and osteoblast phenotypes in combination with high regenerative capacity for the vasculature^7^ and other organs. In bone, MSC can top up the osteoblast pool, thereby increasing bone density and fracture resistance^8^. Both compartments, bone and vasculature, are in close interaction, reciprocally regulated via cellular, endocrine, and metabolic signals^9^. During aging, they progressively lose their adaptability and regenerative capacity on cellular and systemic levels^10,11^. Consequently, deregulated cellular differentiation processes lead to calcium loss from bones and increased calcium deposition in blood vessels. Clinical correlates are osteoporosis and arteriosclerosis^12,13^. Chronic metabolic disease conditions such as diabetes mellitus or chronic kidney disease with uremia accelerate both detrimental clinical phenotypes^14^. This involves mechanisms usually associated with age, such as premature cellular senescence and other cell fates^15^ adversely affecting viability, adaptibility, and resistance to stress^10,11,16^.

Arterial calcification has been attributed to active transformation of VSMC to osteoblast-like cells reminiscent of intramembranous and enchondral bone formation^17,18^. However, cells with an MSC phenotype found in the arterial adventitia have been shown to be a major source of osteoblast-like cells in intimal and medial calcification in a mouse model of chronic kidney disease^19^. The uremic millieu triggers osteoblastic differentiation of MSC and calcification^20^, implying loss of vascular progenitor properties. Hence, preservation and restoration of physiologic MSC function for endogenous regeneration can be considered more effective than targeting terminally differentiated cells such as VSMC and osteoblast-like cells to counteract or even reverse accelerated vascular calcification in patients with pro-arteriosclerotic conditions.

The atypical serine/threonine kinase mechanistic target of Rapamycin (mTOR) orchestrates cellular functions in response to metabolic cues and growth factors^21^. It is contained in two structurally and functionally distinct multi-protein complexes, mTOR complex 1 (mTORC1) and mTOR complex 2 (mTORC2). Activation of both complexes facilitates cell growth and survival^21,22^. The major function of the mTORC1 down-stream targets p70-S6 kinase, 4E-BP1 and others is promotion of cellular growth and proliferation via increased protein and lipid synthesis^23^ and inhibition of autophagy^24^ at the cost of premature cellular senescence^25^. Functions of mTORC2 are less well studied and include organization of the actin cytoskeleton^26^, control of ion transport^27^, and anti-apoptotic properties via stimulation of the AKT-FOXO pathway^28^. Direct involvement of specific branches of the mTOR signaling network in regulation of mammalian life-span^29^, cellular senescence^25,30^, and vascular smooth muscle and osteoblast differentiation^5,6,31,32^ opens a perspective for therapeutic mTOR targeting in aging-related pathologies of the bone-vascular axis. The macrolide Rapamycin (Rapa) is a complex modulator of mTOR signaling since it effectively blocks most but not all mTORC1 functions^21^ without inhibitory effects on mTORC2, aside from a few exceptions^33^. Rapa and its derivatives, the “rapalogs”, are increasingly used in clinical applications ranging from local release in vessels from drug-eluting stents to systemic therapy of cancer, immunologic and genetic diseases as well as immunosuppression after organ transplantation. In animal models, Rapa has been shown to counteract degenerative and age-related pathologies in heart and brain^34,35^, making it a prototypic candidate to stimulate endogenous regenerative processes.

We tested the hypothesis that during osteoblastic transformation of MSC, mTORC1 and mTORC2 orchestrate cell-fate programs in a chronologically coordinated manner resulting in osteoblast differentiation and calcification. In addition, we provide evidence from *in vitro* and *in vivo* studies that enhanced mTORC2 function was both indispensable and sufficient to establish a regenerative cell fate pattern conferring protection from calcification to MSC *in vitro*. Selective induction of protective cell fate programs via therapeutic manipulation of mTOR network components holds promise to preserve and restore a regenerative MSC phenotype capable of rejuvenating the bone-vascular axis in age-related pathologies and to protect from accelerated vascular calcification caused by metabolic diseases.

## Materials and Methods

All experiments were performed in accordance with relevant guidelines and regulations. The isolation of MSC was approved by the Ethik-Kommission der Ärztekammer Hamburg (#2572) and the Ethikkommission Ethikausschuss 4, CBF, Charité (#EA4/114/11). The study was conducted in accordance with the Declaration of Helsinki and was approved by local ethic authorities. All subjects provided written informed consent. The experiments were conducted according to institutional guidelines (“Gute Wissenschaftliche Praxis”, Charité University Hospital). All animal experiments were approved by local authorities (LaGeSo G0028/11, Berlin, Germany) and were conducted according to institutional animal care guidelines (Charité University Hospital).

### Isolation and culture of MSC

MSC were isolated from bone marrow aspirates obtained from 20 healthy bone marrow donors (7 female, 13 male), median age 31 years (range 0.5–42) following a previously described protocol^36^. In brief, bone marrow mononuclear cells were purified by Ficoll density gradient centrifugation. Plastic adherent cells were expanded in α-MEM (#E15-862, PAA) supplemented with penicillin/streptomycin (Gibco), 2 IU/ml heparin (Ratiopharm), and 5% platelet lysate at 37 °C and 5% CO_2_. Cells were used up to passage 5.

### Characterization of MSC

All preparations were assessed for surface marker expression, chondroblastic, adipocytic and osteoblastic differentiation capacity as well as expression of VSMC marker proteins. Three cell preparations were exemplarily studied for functional L-type calcium channels as described below.

50,000 cells were labeled with 3 µl antibody or corresponding isotype control and analyzed using a Beckton Dickinson FACS Calibur. CD73 (BD, #561254), CD90 (Miltenyi, #130-095-403), CD105 (BD, #561443), CD11b (Miltenyi, #130-081-201), CD14 (BD, #557153), CD19 (Miltenyi, #130-091-328), CD34 (Miltenyi, #130-092-213), CD45 (BD, #555492), HLA-DR (BD, #559866), with the corresponding isotype controls IgG2a (BD, #553456), isotype IgG2aκ (BD #555573), and isotype IgG1 (Miltenyi, #130-081-002), were used.

Chondroblastic differentiation was tested in pellet cultures with 1 × 10^6^ cells in 4 ml DMEM containing 1 mM sodium pyruvate (Applichem), 20 mM HEPES (Roth) pH 7.3, 0.1 µM dexamethasone, 0.1 mM 2-phospho-L-ascorbic acid, and 10 ng/ml TGF-β1 (R&D). After 4 weeks, protein extraction and western blot analysis were performed.

To induce adipogenesis, confluent monolayers were treated with DMEM containing 1 μM dexamethasone, 0.01 mg/ml insulin (Berlin-Chemie), 0.2 mM indomethacin (Cayman Chemical Company), and 0.5 mM 3-isobutyl-1-methyl-xanthine (Serva) for 4 weeks. After fixation with 4% paraformaldehyde, staining was performed with 6 ml of 0.5% Oil red O (Sigma) in isopropanol added to 4 ml deionized water.

For osteoblastic differentiation, 13,000 MSC/cm² were incubated with osteoblast induction medium (OM) consisting of Dulbecco’s Modified Eagle’s Medium (DMEM; PAA) supplemented with 2 mM glutamine (Lonza), penicillin/streptomycin (Gibco), 1% FCS (Lonza), 10 mM β-glycerophosphate (Applichem), 500 µM ascorbic acid, and 100 nM dexamethasone (both from Sigma) for 3 weeks. After fixation with ice-cold methanol, cells were stained with filtered 5% Alizarin (1,2-dihydroxyanthraquinone, Sigma) pH 4. After several wash steps with PBS pH 6.0, wells were dried.

Photomicrographs were taken on a Zeiss Axiovert 40 CFL using a Canon PowerShot A649.

### L-Type Ca2+ channel imaging

In six-well-plates, 75,000 cells per well were seeded onto glass coverslips in complete α-MEM and allowed to adhere overnight. Medium was switched to phenol red free DMEM without FCS or platelet lysate for 24 h. Cells were loaded with the Ca2+ indicator fluo-4-AM (Invitrogen, Karlsruhe, Germany) (10 μM) and pluronic acid (Merck, Darmstadt, Germany) (0.01%; w/v) for 30 min at room temperature in PSS (NaCl 134 mM, KCl 6 mM, CaCl2 2 mM, MgCl2 1 mM, HEPES 10 mM, glucose 10 mM, pH 7.4 with NaOH). Before taking records, the cells were washed with PSS and further incubated for 20 min to allow de-esterification of the dye.

Pretreatment with 1 μmol/L nimodipine (Sigma-Aldrich) was carried out for 5 minutes before adding KCl. Fluo-4 loaded cells were imaged using a BioRad MRC 1024 laser scanning confocal microscope attached to a Nikon Diaphot 300 inverted microscope. Excitation was performed at 488 nm and the emission wavelength was 500 nm. Images were collected at a rate of 1/second. Image processing was done using imageJ 1.41i (National Institutes of Health, USA, http://rsbweb.nih.gov/ij/). Background fluorescence was subtracted and changes in intracellular calcium were expressed as relative fluorescence changes, i.e. F/Fo (with Fo indicating the fluorescence before stimulation and F the time-dependent fluorescence signal after stimulation). Peak amplitudes of Ca2+ transients were calculated as (Fpeak-Fo)/Fo. Stock solutions of fluo-4 AM (2.5 mM) and of nimodipine (1 mM) were made using DMSO as solvent. High external potassium solutions were made by iso-osmotic substitution of NaCl with KCl in the PSS.

### Drug treatments

According to the manufacturer’s instructions, the following stock solutions were prepared with DMSO: Rapa (LC Laboratories) 20 µM, Bafilomycin A1 (Selleck) 10 µM, MK-2206 (Selleck) 100 µM. Aliquots were stored at −80 °C. Controls were treated with the equivalent volume of DMSO.

### Immunocytochemistry

13,000 MSC/cm² were seeded on Thermanox Plastic Coverslips (Nunc) in indicated medium for 3 weeks. After fixation with methanol and blocking with 3% BSA/PBS, primary antibodies (Osteopontin, abcam, ab8448; Collagen I, abcam, ab34710; both 1:500 in blocking solution) were incubated for 2 hours at 37 °C in a humid chamber. After washing with PBS, secondary antibody incubation (HRPO-conjugated IgG, Dianova) followed. Signal was developed with AEC High Sensitivity Substrate Chromogen (Dako) before extensive washing.

### Western blot analysis

Western blot was performed following standard protocols. Blocking was done with 10% BSA/TBS-T for 2 hours at room temperature. All antibodies were diluted in blocking solution as follows: SM22alpha (abcam, ab10135, 1:10 000), Calponin (Sigma, 1:10 000, C2687), MLCK (Sigma, 1:500, M7905), SMA (Sigma, 1:5000, M7905), Collagen IIA1 (Santa Cruz, scr-52658, 1:400), Osteopontin (abcam, ab8448, 1:1,000), Cbfa1/Runx2 (MBL, D130-3, 1:500), Osterix (abcam, ab22552, 1:500), Collagen III (abcam, ab6310, 1:500), pp70S6K^Thr389^ (R&D, AF8963, 1:500), pp70S6K^Thr421/Ser424^ (CST, #9204, 1:1,000), pAKT^Ser473^ (CST, #4080, 1:1,000) pAKT^Thr308^ (CST, #2965, 1:1,000), ERK1/2^Thr202/Tyr204^ (CST, #9106, 1:1,000), LC3B (Novus, NB100-2220, 1:1,000), p16^INK4a^ (Santa Cruz, sc-468, 1:500), SQSTM1/p62 (#5114, CST, 1:1000), cleaved caspase 3 (CST, #9661, 1:500), Bcl-2 (Santa Cruz, sc-492, 1:500), Rictor (CST, #9476, 1:1,000), mTOR (CST, #2972, 1:1000), Raptor (CST, #2280, 1:1000), AKT (CST, #9272 1:2000), p70-S6 (CST, #9202, 1:1000), ERK1/2 (CST, #9102, 1:1000), α-Tubulin (Sigma, T9026, 1:6000), GAPDH (hytest, 5G4, 1:100,000). After incubation with secondary antibodies (Dianova), SuperSignal West Chemiluminescent Substrate (Thermo Fisher) was used for visualization in a G:BOX F3 device (Syngene).

### Alkaline phosphatase activity

Activity of alkaline phosphatase (ALP) was determined at day 7 after induction of osteoblast differentiation. Cells were lysed with 250 µl ALP lysis buffer (150 mM Tris pH 10.0 (Roth), 0.1 mM ZnCl_2_, 0.1 mM MgCl_2_, 1% Triton-X100 (all from Applichem)) at room temperature under constant agitation for 30 minutes. Supernatants were centrifuged for 10 min at 12,000 rpm and 4 °C. Each sample was measured in 4 replicates in a 96-well-plate with 50 µl per well mixed with 200 µl substrate solution (ALP buffer with freshly dissolved p-Nitrophenyl phosphate (Fluka) at 2.7 mM). Optical density (OD) at 405 nm was measured at baseline and every 5 min during the incubation time of 1 hour at 37 °C. ∆OD values to baseline ODs at one chosen time point during the linear phase were divided by the protein concentration of the sample determined with the DC Protein Assay (Bio-Rad).

### Calcium deposition

Extracellular calcium deposition of differentiating MSC was assessed after 3 weeks of incubation with OM. Calcium was solubilized by shaking cells overnight in 200 µL 0.6 M HCl at 4 °C. Samples were centrifuged for 60 min at 20,000× g and 4 °C. 10 µL of either calcium standards or sample supernatant were mixed with 150 µL 0.1 mg/mL ortho-cresolphthalein complexone, 1 mg/mL 8-hydroxy-quinoline, 0.7 M HCl, and 150 µl 15% 2-amino-2-methyl-1-propanol in H_2_O, pH 10.7. OD was measured at 540 nm in duplicates. Blank absorption was subtracted and calcium concentrations were calculated using a standard curve. To analyze the protein content for normalization, 230 µl 0.1 M NaOH/0.1% SDS solution was added to the remainder of the sample and mixed for about 10 minutes. After centrifugation at 20,000× g for 10 min, 20 µl were used for protein quantification with the DC protein assay (Bio-Rad) in duplicates.

### X-Gal staining

Senescence was visualized via hydrolysis of 5-bromo-4-chloro-3-indolyl-β-D-galactopyranoside by cellular β-galactosidase. MSC were seeded on glass slides in 12-well plates at 50,000 cells per well and incubated with indicated medium for 3 weeks. Cells were fixed in 2% paraformaldehyde/0.25% glutaraldehyde in PBS with 1 mM MgCl_2_ pH 6.0. Staining was performed for 12 hours at 37 °C using 5 mM K_3_Fe(CN)_6_/5 mM K_4_Fe(CN)_6_ × 3H_2_O in PBS with 1 mM MgCl_2_ pH 6.0 freshly supplemented with 40 mg 5-Bromo-4-chloro-3-indolyl-β-D-galactoside/ml and N,N-Dimethylformamide to a final concentration of 1 mM. Coverslips were washed and mounted on glass slides with AquaPolymount (Polyscience Inc) before micrographs were taken.

### Apoptosis ELISA

Quantification of apoptosis was performed by measuring fragmented DNA with the Cell Death Detection ELISA PLUS Kit (Roche) following the manufacturer’s instructions. To normalize for protein content, total protein of lysates was quantified with the DC protein assay (Bio-Rad).

### LDH activity

LDH analysis was performed with the Cytotoxicity Detection Kit (Roche) following the manufacturer’s instructions. To normalize for protein content, 500 µl cell culture supernatant were mixed with 250 µl 20% trichloric acid, incubated on ice for 30 min, and centrifuged for 30 min at 4 °C with 20,000× g. The pellet was washed twice with ice-cold acetone. After drying, the pellet was resolved in protein lysis buffer (100 mM Tris pH 8,0; 0.2% SDS; 1% Triton X-100), and total protein was quantified with the DC protein assay (Bio-Rad).

### Cloning and lentivirus production

For rictor knockdown, pSuperRetroPuroshRNA-Rictor and pLVTH were digested with EcoRI/ClaI and the shRNA-fragment was ligated into the pLVTH-backbone. For flag-rictor over-expression, the pLJM1 vector-backbone was used. After confirmation of plasmid accuracy, lentiviral particles were produced with a second generation packaging system in 293T cells via calcium phosphate-transfection. Virus-production medium containing 10% FCS and 1.2% BSA was collected 24 and 48 hours later. After straining through 0.45 µm filters and concentrating via ultracentrifugation (2 hours at 100,000× g), virus was supplemented with protamine sulfate (final concentration 10 µg/ml) before MSC were transduced at low cell density. Efficiency was confirmed by GFP expression.

### Animals

All experiments were approved by local authorities (LaGeSo G0028/11, Berlin, Germany) and were conducted according to institutional animal care guidelines (Charité University Hospital). As previously described^37^, male ten-week-old mice (C57BL/6JRccHsd, Harlan Winkelmann) were exposed to Rapa (1.5 mg/kg) or vehicle, both administered intraperitoneally every third day over a period of 37 days. Rapa was dissolved in DMSO and diluted 1:1000 in a mixture of medium chain triglycerides (MCT; Miglyol 812, Caesar & Lorentz). Vehicle consisted of DMSO diluted 1:1000 in MCT. The administered volume was 5 mL/kg resulting in 0.1–0.15 mL per injection depending on the individual body weight of the mice. Aortas were excised, snap frozen in liquid nitrogen, and stored at −80 °C until analysis. 4 mice per group were studied.

### Immunofluorescence histology

Cryosections of 6 µm were prepared using Tissue-Tek ® O.C.T™ Compound (Sakura) and a CM1900 Leica Cryostat. After fixation, slides were blocked for 4 hours with 5% rabbit serum (Invitrogen). Primary antibodies were incubated for 12 hours as follows: pAKT^Ser473^ (CST, #4080, 1:200), and pp70-S6^Thr389^ (Santa Cruz, sc-11759, 1:100) in 3% rabbit serum-PBS. Bcl-2 (Santa Cruz, sc-492, 1:250), p16^INK4a^ (Santa Cruz, sc-468, 1:250), caspase 3 (Santa Cruz, sc-7148, 1:250), and LC3B (Novus, NB100-2220, 1:250) in 3% rabbit serum-TBS-T with 0.1% Triton-X100 (Applichem). Anti-rabbit and anti-goat Alexa Fluor 586 secondary antibodies (Invitrogen, 1:2000) were applied in TBS-T with 1% goat serum for 2 hours at room temperature. Micrographs were taken using an Axiacam HR camera device on an Axio Imager A1 immunofluorescence microscope (Zeiss). For quantification, 100 DAPI stained nuclei were counted and the proportion of antibody positive cells was determined.

### Statistics

All data are expressed as mean +/− SEM and were analyzed with 1-way or 2-way ANOVA followed by post-testing with Bonferroni’s test for multiple comparisons as appropriate. All analyses were performed with GraphPad Prism version 5.02 for Windows (GraphPad Software, San Diego California USA). Significance was considered at a value of p < 0.05.

## Results

### Cell culture model for osteoblastic differentiation of MSC

**Figure 1:**
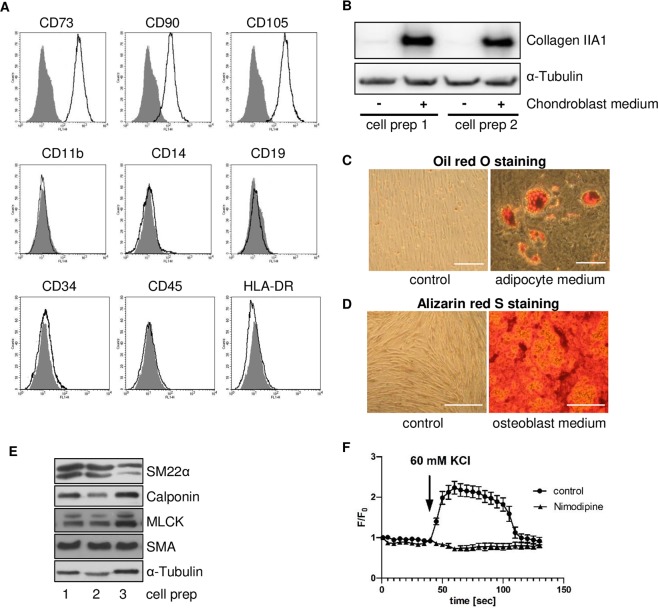
Cell culture model for osteoblastic differentiation of pluripotent human bone marrow-derived MSC. (**A**) Representative FACS analysis out of 20 of an MSC defining surface marker panel confirming homogeneity of isolated cells. More than 98% of cells are positive for CD73, CD90 and CD105 and negative for CD11b, CD14, CD19, CD34, CD45, and HLA-DR compared to isotype control (grey). (**B**) Chondroblastic differentiation of MSC after incubation for 32 days with chondroblast induction medium demonstrated by western blot analysis for chondrocyte-specific type II collagen. Two representative cell preparations out of 20 are shown. α-Tubulin served as loading control. (**C**) Adipocytic differentiation of MSC after incubation for 21 days with adipocyte induction medium demonstrated by staining of intracellular lipid droplets with Oil red O. Representative experiment out of 20, phase contrast microscopy, original magnification × 200, scale bar = 50 µm. (**D**) Osteoblastic differentiation of MSC after incubation for 21 days with osteoblast induction medium demonstrated by staining of extracellular hydroxyapatite deposits with Alizarin red S. Representative experiment out of 20, phase contrast microscopy, original magnification x100, scale bar = 50 µm. (**E**) Baseline expression of vascular smooth muscle marker proteins in MSC: SM22α, smooth muscle calponin (sm-Calponin), myosin light chain kinase (MLCK), smooth muscle-α-actin (SMA). Western blot analyses of three representative cell preparations out of 20 are shown. α-Tubulin served as loading control. (**F**) Calcium transients without (control) and after pretreatment with nimodipine (Nimodipine) in MSC at base line showing expression of functional voltage-dependent dihydropyridine-sensitive (Ca_v_1.2) L-type Ca^2+^ channels. Representative experiment out of three; 20–30 individual cells were studied for each tracing.

To study mTOR-dependent cell fate programs during osteoblastic differentiation in progenitor cells, a cell culture model using human bone marrow-derived MSC with characteristic cell surface marker profile and multilineage differentiation capacity as defined by the International Society for Cellular Therapy^38^ was established (Fig. 1A–D). In addition, MSC displayed features of vascular smooth muscle cells (VSMC) as previously reported^5,6,39^. MSC expressed the smooth muscle marker proteins SM22α, smooth muscle calponin (sm-Calponin), myosin light chain kinase (MLCK) and smooth muscle-α-actin (SMA) (Fig. 1E). Moreover, they featured functional voltage-dependent dihydropyridine-sensitive (Cav1.2) L-type calcium channels characteristic for VSMC (Fig. 1F).

**Figure 2:**
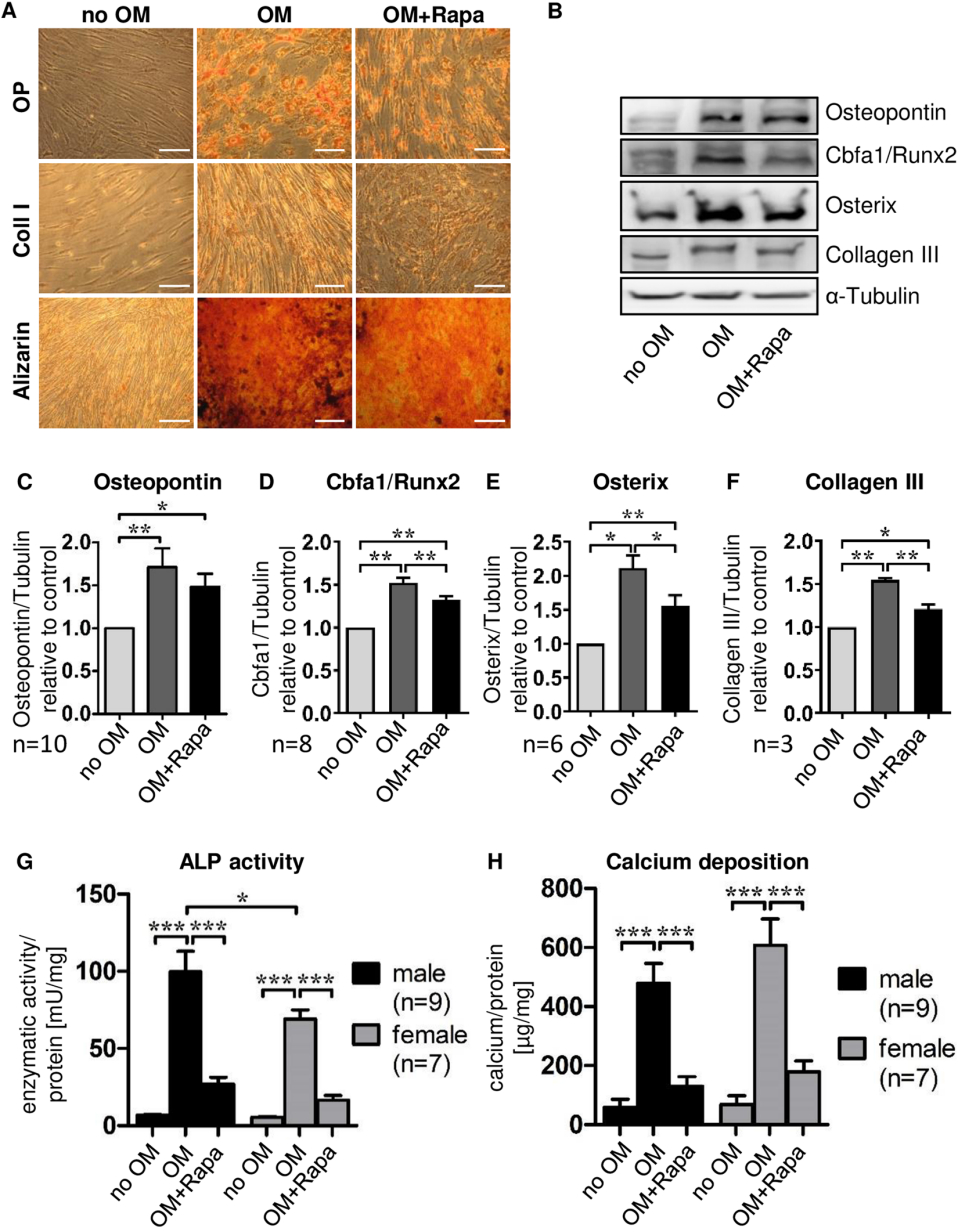
Modulation of MSC osteoblastic differentiation by Rapa. MSC were cultured under control conditions (no OM) or with osteoblast induction medium in presence of vehicle (OM) or 20 nM Rapa (OM+Rapa). (**A**) Immunocytochemistry for osteoblast marker proteins and Alizarin red S staining of calcium deposits after 21 days. Osteopontin (OP), collagen I (Coll I). Representative experiment out of 20, phase contrast microscopy, original magnification × 200, scale bar  =  100 µm. (**B**–**F**) Western blot analysis of osteoblast marker proteins. MSC were cultured for 21 days under control conditions (no OM) or with osteoblast induction medium in the presence of vehicle (OM) or 20 nM Rapa (OM+Rapa). Representative western blots (**B**) and densitometric quantifications (**C**–**F**) are shown. Numbers of independent experiments are indicated at the bottom of each graph. Band intensities were normalized to α-tubulin as a loading control. Control (no OM) was set to 1. Bars represent mean + SEM, *P < 0.05, **P < 0.01, ***P < 0.001. (**G**) Alkaline phosphatase (ALP) activity was measured after 7 days of incubation and normalized to total protein concentrations of lysates. Cells from 9 male and 7 female individuals were analyzed. (**H**) Calcium deposition was quantified after incubation for 21 days and normalized to total protein concentrations. Cells from 9 male and 7 female individuals were analyzed.

Phenotype conversion was induced by incubation with osteoblast induction medium (OM) containing high phosphate concentrations for 21 days. MSC derived osteoblast-like cells expressed the osteoblast marker proteins osteopontin, collagen I, Cbfa1, osterix, and collagen III (Fig. 2A–F) and displayed high levels of alkaline phosphatase (ALP) activity (Fig. 2G). On a functional level, differentiated cells extensively produced calcified extracellular matrix (Fig. 2A,H). MSC from male donors developed slightly higher ALP activity than MSC from female donors after osteoblastic differentiation (Fig. 2G) while calcium deposition did not differ significantly (Fig. 2H).

### The mTOR network controls osteoblastic differentiation of MSC

The involvement of mTOR signaling in osteoblastic phenotype conversion was tested with the mTOR inhibitor Rapa. Cells co-incubated with Rapa throughout the whole differentiation period of 21 days expressed lower levels of osteoblast marker proteins (Fig. 2A–F), and displayed strongly reduced ALP activity after 7 days (Fig. 2G) and calcium deposition after 21 days (Fig. 2H), indicating a central role for mTOR in osteoblastic differentiation of MSC. There was no interaction between sex and the response to treatment (Fig. 2G,H).

**Figure 3:**
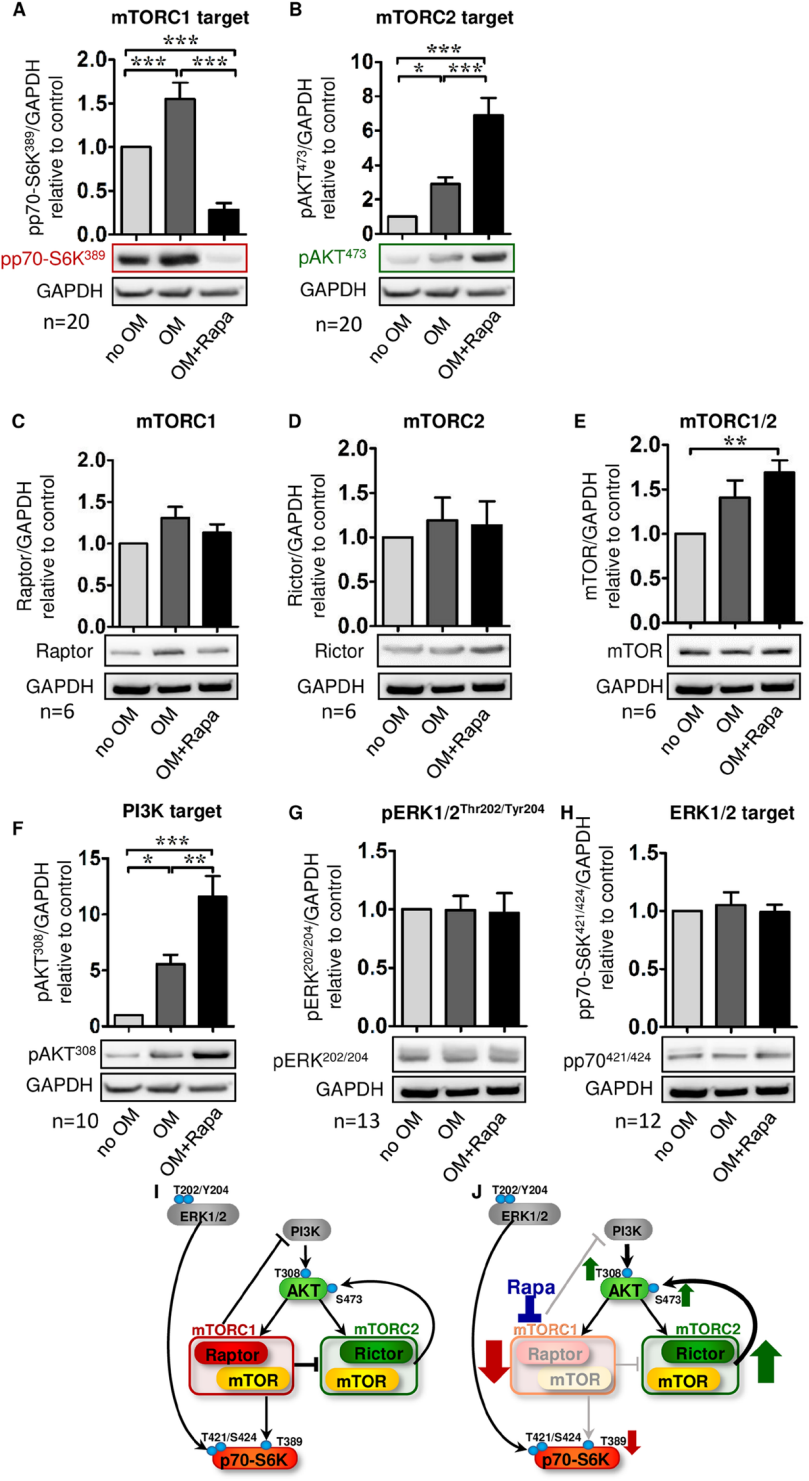
Western blot analysis of the mTOR network during osteoblastic differentiation of MSC. Cells were cultured for 21 days under control conditions (no OM) or with osteoblast induction medium in presence of vehicle (OM) or 20 nM Rapa (OM+Rapa). (**A**) Phosphorylation of p70-S6 kinase at threonine 389 (pp70-S6K^389^) for activation of mTORC1. (**B**) Phosphorylation of AKT at serine 473 (pAKT^473^) for activation of mTORC2. (**C**) Raptor as the key subunit of mTORC1. (**D**) Rictor as the key subunit of mTORC2. (**E**) mTOR as the serine/threonine kinase component of mTORC1 and mTORC2. (**F**) Phosphorylation of AKT at threonine 308 (pAKT^308^) as an upstream modulator and part of a feedback loop involving phosphatidyl inositol-3 phosphate kinase (PI3K). (**G**) Phosphorylation of ERK1/2 at threonine 202/tyrosine 204 (pERK^202/204^), and (**H**) its downstream target p70-S6 kinase at threonine 421/serine 424 (pp70-S6K^421/424^) as a side branch of p70-S6K activation. Densitometric quantifications and representative western blots are shown. Numbers of independent experiments are indicated at the bottom of each graph. Band intensities were normalized to GAPDH as a loading control. Control (no OM) was set to 1. Bars represent mean + SEM, *P < 0.05, **P < 0.01, ***P < 0.001. (**I**) Scheme of the mTOR network without, and (**J**) with modulation by Rapa.

As part of a detailed analysis of the mTOR network, activation of the two distinct signaling branches, mTORC1 and mTORC2, was analyzed in osteoblasts derived from MSC in comparison to undifferentiated MSC. MSC-osteoblasts differentiated for 21 days displayed about 50% higher phosphorylation levels of p70-S6 kinase at threonine 389 (p70-S6K; Fig. 3A), a direct downstream target of mTORC1 (Fig. 3I,J). Phosphorylation of AKT at serine 473, downstream of mTORC2 (Fig. 3I,J), was increased 3-fold (Fig. 3B). Interestingly, Rapa intervention potently reduced mTORC1-dependent p70-S6K phosphorylation in MSC-osteoblasts (Fig. 3A), while mTORC2 activity was tremendously upregulated (Fig. 3B). Expression levels of raptor and rictor, the two key proteins forming mTORC1 (raptor) and mTORC2 (rictor) in association with mTOR were not changed upon treatment with OM alone or with OM and Rapa (Fig. 3C,D). Levels of mTOR increased slightly when MSC where treated with OM and Rapa (Fig. 3E). Investigation of upstream modulators and feedback loops of mTOR complexes revealed activation of phosphatidyl inositol-3 phosphate kinase (PI3K) signaling in MSC-derived osteoblasts as evidenced by phosphorylation of AKT at threonine 308 (Fig. 3F). Rapa treatment further enhanced PI3K activity (Fig. 3F). Extracellular regulated kinase 1/2 (ERK1/2) and the ERK-dependent phosphorylation site of p70-S6K (pp70-S6K^Thr421/Ser424^) did not show altered phosphorylation after 3 weeks of OM stimulation without or with Rapa (Fig. 3G,H). These findings indicate that the osteoblastic phenotype of MSC is associated with signaling via PI3K and its downstream targets mTORC1 and mTORC2 (Fig. 3I). Rapa-mediated prevention of osteoblastic transformation and calcification is related to inhibition of mTORC1 with simultaneous mTORC2 activation (Fig. 3J). The stimulation of mTORC2 by Rapa can be explained by release of direct inhibitory signals from mTORC1 on mTORC2 and on PI3K (Fig. 3I,J)^40^.

### Degenerative cell fates are activated during osteoblastic differentiation of MSC in a temporally coordinated manner

**Figure 4:**
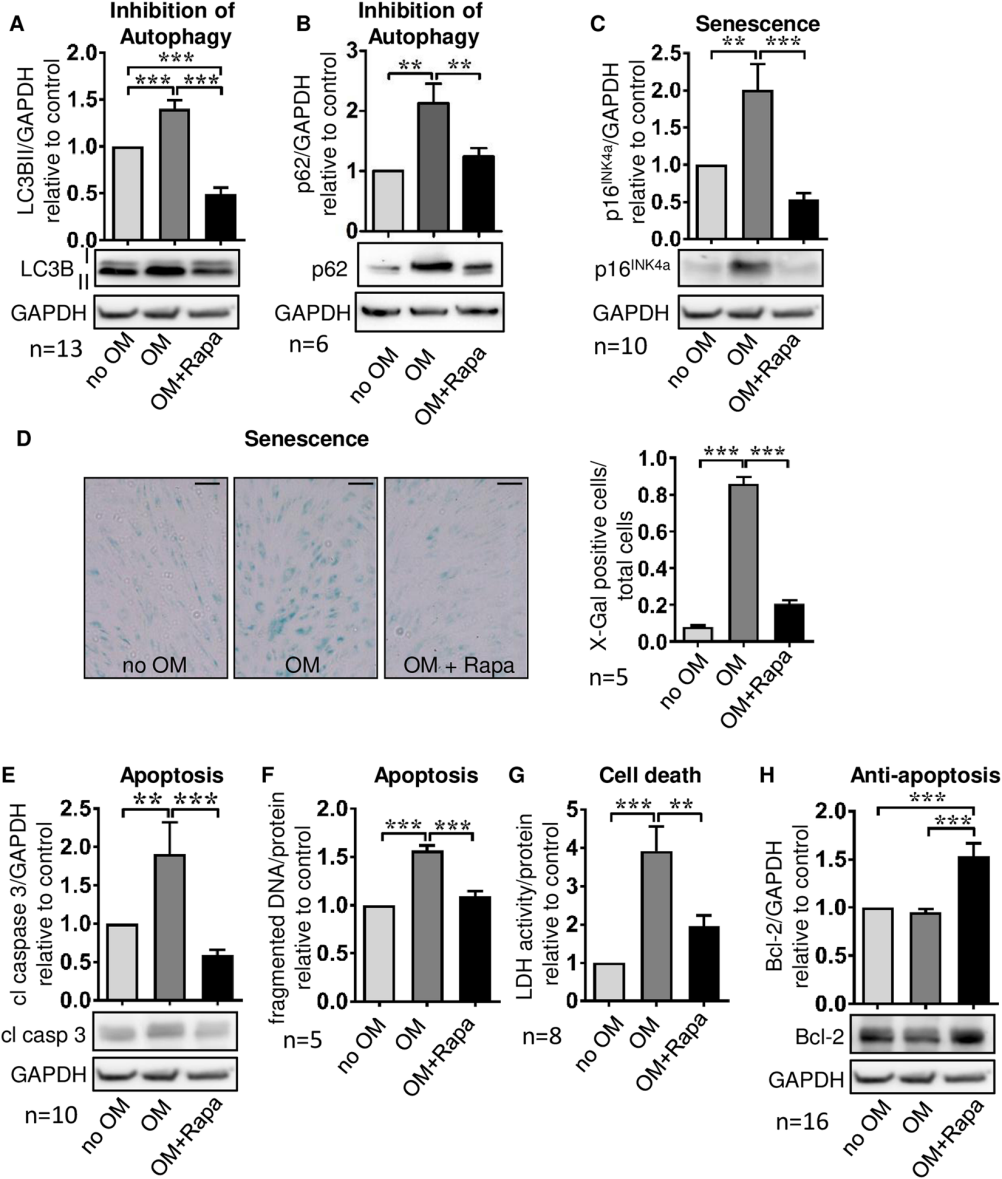
Degenerative cell fates are activated during osteoblast differentiation of MSC. Cells were cultured for 21 days under control conditions (no OM) or with osteoblast induction medium in presence of vehicle (OM) or 20 nM Rapa (OM+Rapa). (**A**) Western blot analysis of LC3B II and (**B**) of p62 for autophagic flux with higher levels indicating less degradation due to reduced autophagic flux. (**C**) Western blot analysis of p16^INK4a^ indicating cellular senescence. (**D**) X-Gal staining for assessment of β-galactosidase a marker of cellular senescence. Representative experiment out of five, phase contrast microscopy, original magnification x400, scale bar = 100 µm. (**D**) Western blot analysis of cleaved caspase 3 to test for apoptosis. (**E**) ELISA measuring fragmented DNA normalized to total protein concentrations to quantify apoptosis. (**F**) Lactate dehydrogenase (LDH) activity in cell culture supernatants normalized to total protein concentrations for quantification of cell death. (**G**) Western blot analysis of the anti-apoptotic protein Bcl-2. Numbers of independent experiments are indicated at the bottom of each graph. For all western blot analyses representative blots are shown. For densitometric quantification, band intensities were normalized to GAPDH as a loading control. Control (no OM) was set to 1. Bars represent mean + SEM, **P < 0.01, ***P < 0.001.

To establish a mechanistic link between signal and phenotype, cell fate programs related to mTORC1 (autophagy, cellular senescence) and mTORC2 (apoptosis) were studied. The autophagosome constituent LC3B II was more abundant in MSC differentiated into osteoblasts by exposure to OM over 3 weeks (Fig. 4A). In addition, p62, a substrate of autophagosomal degradation, accumulated simultaneously (Fig. 4B). This suggests reduced autophagic flux with less degradation of autophagosomal proteins due to inhibition of autophagy. Osteoblasts also featured high expression levels of p16^INK4a^ (Fig. 4C) and β-galactosidase (Fig. 4D), both indicators of cellular senescence. Increased levels of cleaved caspase 3, fragmented DNA, and LDH released into the supernatant (Fig. 4E–G) in parallel with unaffected levels of the anti-apoptotic protein Bcl-2 (Fig. 4H) indicated apoptotic cell death. MSC that were treated with Rapa during exposure to OM differed markedly in their cell fate responses: Autophagic flux appeared to be enhanced, as amounts of LC3B II and p62 decreased as compared to OM treatment (Fig. 4A,B). Rapa conferred resistance to induction of cellular senescence as indicated by reduced p16^INK4a^ and β-galactosidase (Fig. 4C,D), and apoptosis was prevented as shown by reduced cleaved caspase 3, less fragmented DNA, lower LDH activity in supernatants, and increased Bcl-2 (Fig. 4E–H).

**Figure 5:**
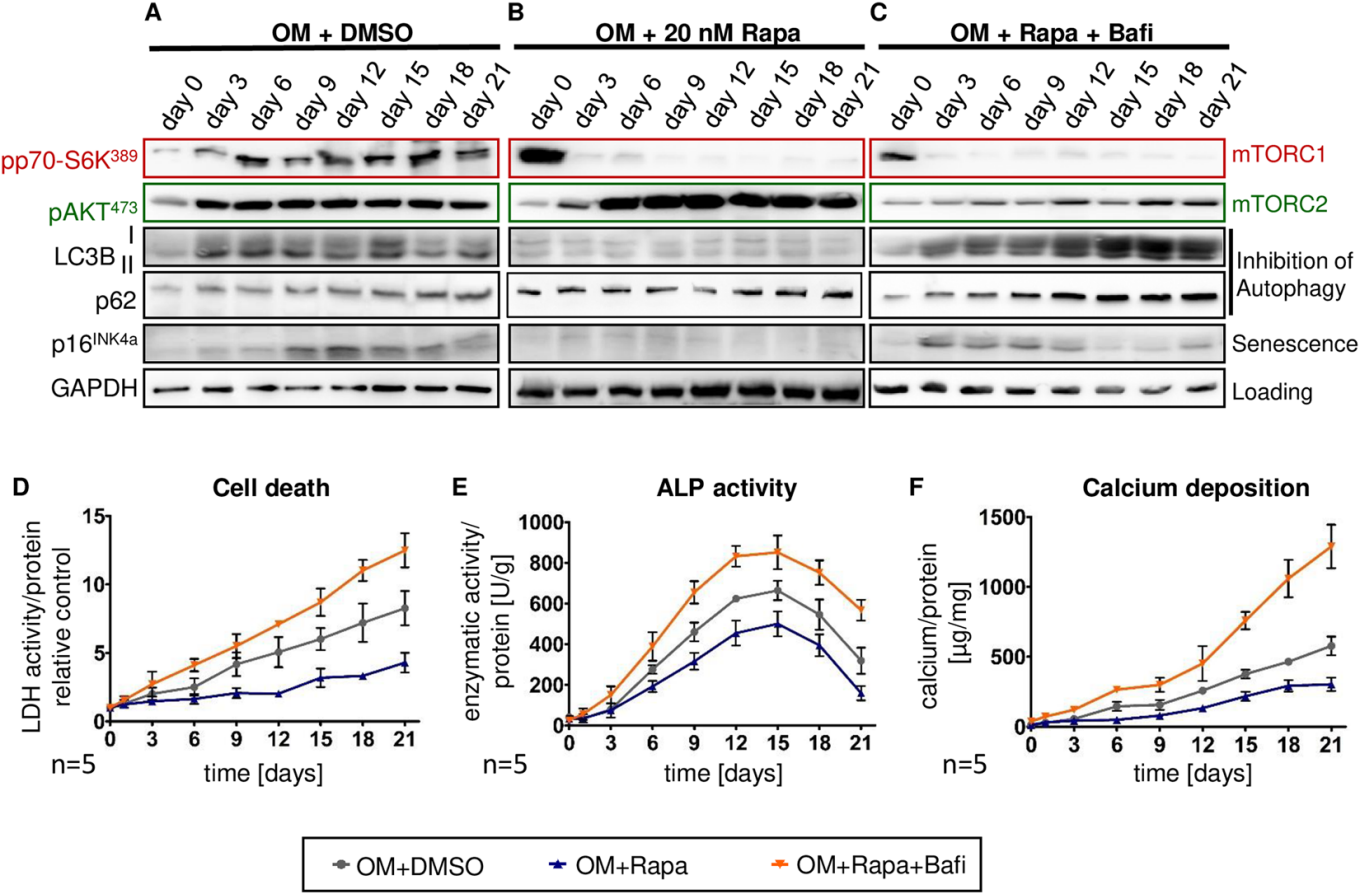
Temporal relations of mTORC1 and mTORC2 signaling, cell fates, and phenotypic changes during osteoblast differentiation. MSC were cultured for 21 days with osteoblast induction medium and vehicle (OM+DMSO), OM and 20 nM Rapa (OM+20 nM Rapa), or OM with 20 nM Rapa and 1 nM bafilomycin A1 (OM+Rapa+Bafi). (**A**–**C**) Representative western blot analyses out of three independent experiments assessing activation of downstream targets of the two mTOR complexes (mTORC1: pp70-S6K^Ser389^; mTORC2: pAKT^Ser473^) and cell fates (Inhibition of autophagy: LC3B II and p62 with higher levels indicating less degradation due to reduced autophagic flux; cellular senescence: p16^INK4a^) at indicated time points. GAPDH serves as loading control. (**D**) Lactate dehydrogenase (LDH) activity in cell culture supernatants normalized to total protein concentrations was determined to quantify cell death. Day 0 was set to 1. (**E**) Alkaline phosphatase (ALP) activity was normalized to total protein concentrations of MSC lysates. (**F**) Calcium deposition was quantified with the ortho-cresolphthalein method and normalized to total protein concentrations. All graphs show mean±SEM, n = 5.

Temporal relations of signaling events, cell fates, and phenotypic changes were deciphered by following MSC undergoing osteoblastic differentiation over a time course of 3 weeks. The earliest events were activation of mTORC1 and mTORC2 detectable on day 3 continuing until the end of the study (Fig. 5A). LC3B II and p62 increased from day 3 onward as an indication of reduced autophagic flux (Fig. 5A). Induction of cellular senescence detected by p16^INK4a^ was first evident on day 9 (Fig. 5A). At the same time, LDH activity in the supernatant started to rise as a sign of cell death (Fig. 5D). Phenotypically, ALP activity, an early marker of osteoblastic cell specificity, rose from day 3 to day 15 and declined thereafter (Fig. 5E). Calcium deposition was first noted on day 6 and further accumulated from day 9 until the end of the study (Fig. 5F). This sequence suggests that signaling via mTORC1 and mTORC2 initiates osteoblastic differentiation indicated by ALP expression. Reduced autophagy precedes cellular senescence and cell death with calcification being the ultimate consequence of these events. ALP activity declines as cells progressively calcify or die.

When Rapa was added, activity of mTORC1 was suppressed far below baseline levels (Fig. 5B) while activation of mTORC2 was prominently increased (Fig. 5B). Accumulation of LC3B II and p62 as well as expression of p16^INK4a^ were almost completely prevented (Fig. 5B). LDH activity showed a delayed (between days 12 and 15) and weak increase (Fig. 5D). ALP activity levels were lower overall (Fig. 5E) while calcium deposition started later (day 12) and was less pronounced (Fig. 5F). This indicates that intervention with Rapa modifies mTOR signaling and potently prevents induction of degenerative cell fates and calcification.

### Blockade of autophagy exacerbates calcification of differentiated MSC

A pharmacologic inhibitor of autophagosomal acidification and degradation, bafilomycin A1, was continuously applied at a low dose (1 nM) to MSC undergoing osteoblastic differentiation in the presence of Rapa to exclude that lower levels of LC3B II in Rapa-treated MSC were due to reduced formation of autophagosomes instead of enhanced autophagic flux. With bafilomycin A1, LC3B II accumulated over time starting as early as day 3 (Fig. 5C), confirming persistent formation of autophagosomes. Levels of p62 increased in parallel as an indication of reduced autophagosomal activity (Fig. 5C). Inhibition of autophagy with bafilomycin A1 profoundly influenced cell fates while mTOR signaling was unaffected (Fig. 5C). Cellular senescence (p16^INK4a^) was greatly induced, even at the earliest time points (Fig. 5C). LDH activity indicating cell death increased with a steeper slope from day 3 until the end of the study (Fig. 5D). With regard to osteoblastic differentiation, ALP enzymatic activity was elevated throughout the whole time course (Fig. 5E). More calcium was deposited from day 3 to day 9 and a remarkably sharp increase started on day 12 resulting in excessive calcium deposition on day 21 (Fig. 5F).

### Inhibition of AKT signaling or genetic depletion of mTORC2 abrogate the protective effect of Rapa on MSC calcification

**Figure 6:**
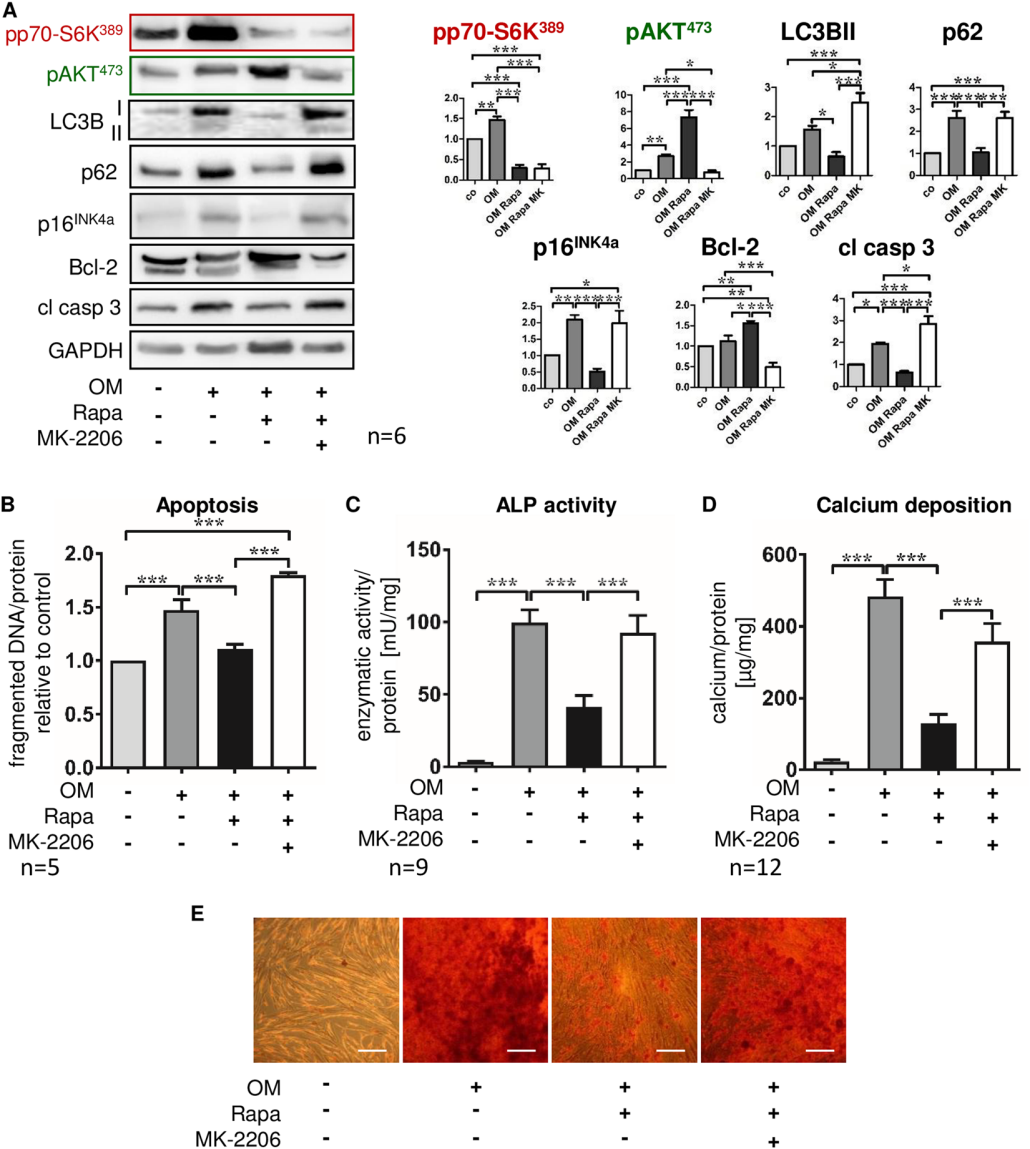
Pharmacologic inhibition of AKT signaling abrogates the protective Rapa effect on MSC calcification. Cells were cultured for 21 days under control conditions, with osteoblast induction medium (OM) and vehicle, OM and 20 nM Rapa, or OM with 20 nM Rapa and the total AKT inhibitor MK-2206 (100 nM) as indicated. (**A**) Western blot analyses assessing activation of downstream targets of the two mTOR complexes (mTORC1: pp70-S6K^Ser389^; mTORC2: pAKT^Ser473^), inhibition of autophagy (LC3B II, p62), cellular senescence (p16^INK4a^), and apoptosis-related proteins (Bcl-2: negative regulator of apoptosis; cleaved caspase 3 (cl casp 3): executioner caspase). Representative western blots from six independent experiments are shown. For densitometric quantification, band intensities were normalized to GAPDH as a loading control. Control (no OM) was set to 1. (**B**) ELISA for fragmented DNA normalized to total protein concentrations to quantify apoptosis. Control was set to 1. (**C**) Alkaline phosphatase (ALP) activity normalized to total protein concentrations was measured after 7 days of incubation. (**D**) Calcium deposition was quantified with the ortho-cresolphthalein method and normalized to total protein concentrations. Bars represent mean + SEM, *P < 0.05, **P < 0.01, ***P < 0.001. Numbers of independent experiments are indicated at the bottom of each graph. (**E**) Alizarin red S staining of calcium deposits. Representative experiment out of six, phase contrast microscopy, original magnification x200, scale bar = 100 µm.

We hypothesized that, in addition to activation of autophagy, prevention of apoptosis is a major mechanism by which Rapa exerts its anti-calcifying effects, since apoptotic bodies have been shown to function as a nidus for incipient calcification processes in VSMC^41^. We employed the pharmacologic AKT inhibitor MK-2206 to block Rapa-enhanced anti-apoptotic signaling via mTORC2/AKT in MSC undergoing osteoblast differentiation. Addition of MK-2206 neutralized amplification of mTORC2-dependent AKT^Ser473^ phosphorylation when mTOR signaling was modulated by Rapa (Fig. 6A). As in the previous experiments, Rapa stimulated autophagic flux as indicated by reduced LC3B II and p62 (Fig. 6A), prevented the induction of cellular senescence (p16^INK4a^, Fig. 6A), enhanced anti-apoptotic mechanisms (Bcl-2, Fig. 6A), and decreased apoptosis as shown by reduced cleaved caspase 3 (Fig. 6A) and fragmented DNA (Fig. 6B). This prevented MSC from osteoblastic transformation and calcium deposition (Fig. 6C-E). When MK-2206 was added in parallel, the protective effects of Rapa were abolished (Fig. 6A–E).

**Figure 7:**
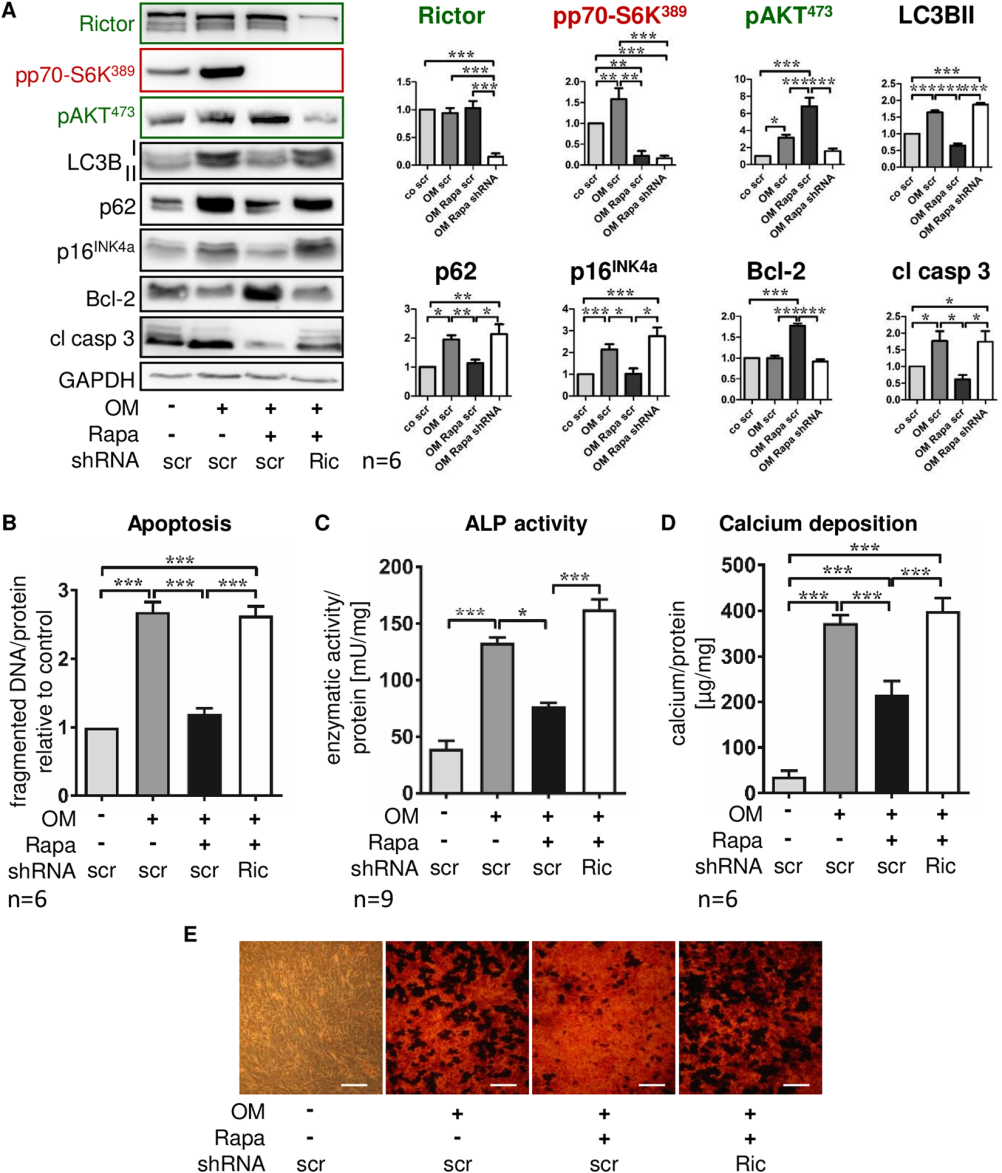
Knock-down of the mTORC2 constituent rictor abrogates the protective Rapa effect on MSC calcification. Unspecific control-shRNA (scramble, scr) or shRNA targeting rictor (Ric) were introduced into MSC by lentiviral transfer. Cells were cultured for 21 days under control conditions, with osteoblast induction medium (OM) and vehicle, or OM and 20 nM Rapa as indicated. (**A**) Western blot analyses of rictor confirming effective knock-down by shRNA, downstream targets of the two mTOR complexes (mTORC1: pp70-S6K^Ser389^; mTORC2: pAKT^Ser473^), inhibition of autophagy (LC3B II, p62), cellular senescence (p16^INK4a^), and apoptosis-related proteins (Bcl-2: negative regulator of apoptosis; cleaved caspase 3 (cl casp 3): executioner caspase). Representative western blots from six independent experiments are shown. For densitometric quantification, band intensities were normalized to GAPDH as a loading control. Control (no OM) was set to 1. (**B**) ELISA for fragmented DNA normalized to total protein concentrations to quantify apoptosis. Control was set to 1. (**C**) Alkaline phosphatase (ALP) activity normalized to total protein concentrations was measured after 7 days of incubation. (**D**) Calcium deposition was quantified with the ortho-cresolphthalein method and normalized to total protein concentrations. Bars represent mean + SEM, *P < 0.05, **P < 0.01, ***P < 0.001. Numbers of independent experiments are indicated at the bottom of each graph. (**E**) Alizarin red S staining of calcium deposits. Representative experiment out of six, phase contrast microscopy, original magnification x200, scale bar = 100 µm.

To test the hypothesis that mTORC2 was the decisive upstream regulator of AKT-dependent anti-calcifying cell fate programs induced by Rapa, we performed lentivirus-mediated shRNA knock-down of the mTORC2-constituting protein rictor. Lentiviral transfection of MSC with targeted or scrambled shRNA was successful as shown by expression of GFP that was also part of the constructs in infected cells (data not shown). Rictor protein expression was reduced by 75% after shRNA transfer (Fig. 7A). While mTORC1 signaling was not influenced by rictor knock-down (pp70S6K^Thr389^; Fig. 7A), Rapa could no longer induce mTORC2 activation when rictor was depleted (pAKT^Ser473^; Fig. 7A). As a result, Rapa-induced reduction of cellular senescence and apoptotic cell death was not detectable when mTORC2 signaling was restricted (Fig. 7A,B). Furthermore, autophagic flux could not be stimulated by Rapa when mTORC2 was deactivated (Fig. 7A). In Rapa-treated MSC undergoing osteoblastic differentiation, knock-down of rictor completely antagonized the reduction of ALP activity (Fig. 7C) and calcium deposition (Fig. 7D,E) observed in MSC with intact rictor expression.

### Enhanced mTORC2 signaling is sufficient to protect MSC from calcification

**Figure 8:**
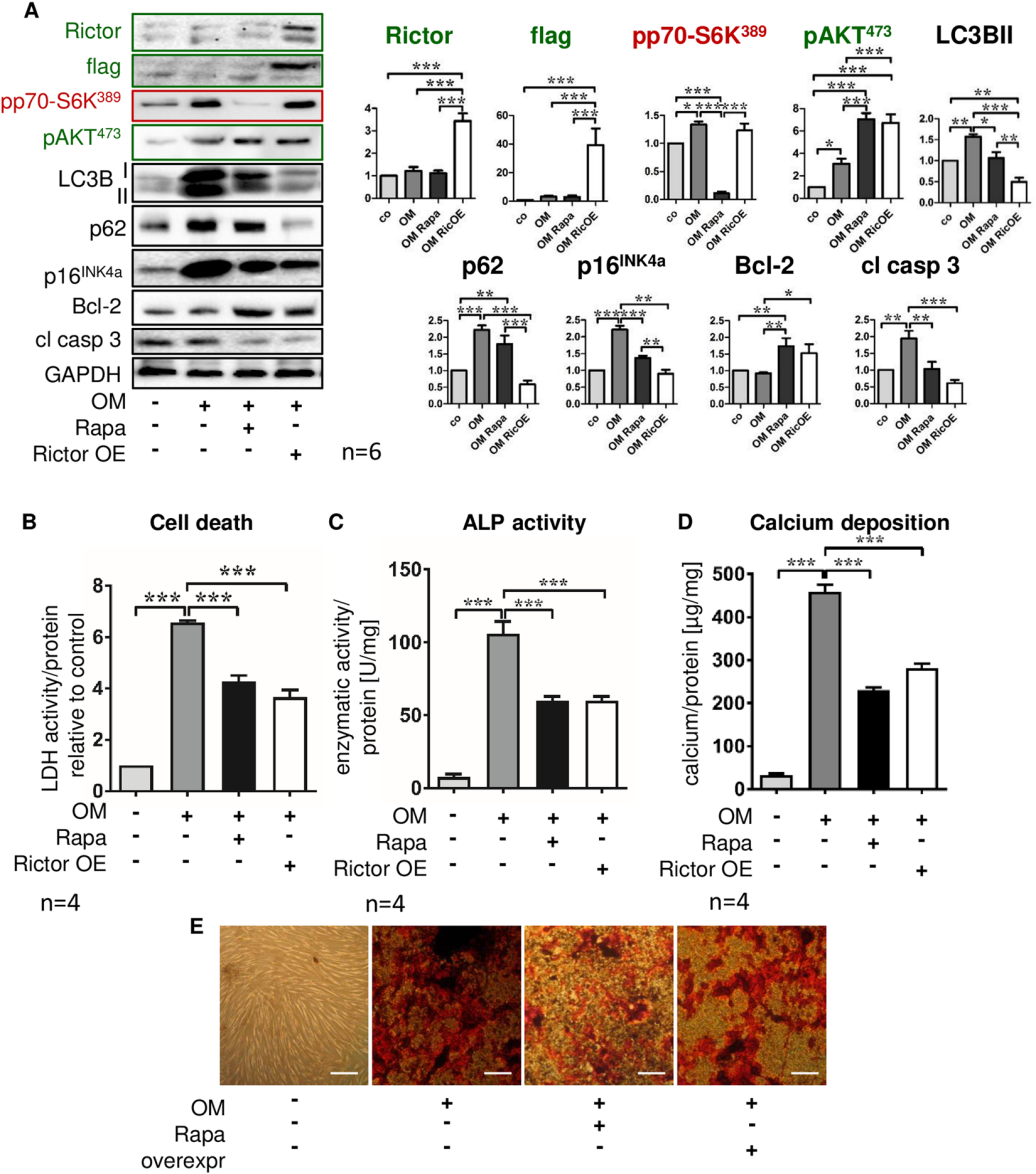
Enhanced mTORC2 signaling is sufficient to protect MSC from calcification. Flag-tagged rictor or green fluorescent protein (GFP) as a control were introduced into MSC by lentiviral transfer. Cells were cultured for 21 days under control conditions, with osteoblast induction medium (OM) and vehicle, or OM and 20 nM Rapa as indicated. (**A**) Western blot analyses of rictor and flag confirming overexpression of rictor due to gene transfer, downstream targets of the two mTOR complexes (mTORC1: pp70-S6K^Ser389^; mTORC2: pAKT^Ser473^), inhibition of autophagy (LC3B II, p62), cellular senescence (p16^INK4a^), and apoptosis-related proteins (Bcl-2: negative regulator of apoptosis; cleaved caspase 3 (cl casp 3): executioner caspase). Representative western blots from six independent experiments are shown. For densitometric quantification, band intensities were normalized to GAPDH as a loading control. Control (no OM) was set to 1. (**B**) Lactate dehydrogenase (LDH) activity in cell culture supernatants normalized to total protein concentrations was determined to quantify cell death. Control was set to 1. (**C**) Alkaline phosphatase (ALP) activity normalized to total protein concentrations was measured after 7 days of incubation. (**D**) Calcium deposition was quantified with the ortho-cresolphthalein method and normalized to total protein concentrations. Bars represent mean + SEM, *P < 0.05, **P < 0.01, ***P < 0.001. Numbers of independent experiments are indicated at the bottom of each graph. (**E**) Alizarin red S staining of calcium deposits. Representative experiment out of six, phase contrast microscopy, original magnification x200, scale bar = 100 µm.

To assess whether or not activation of mTORC2 without inhibition of mTORC1 is sufficient to protect MSC from osteoblastic transformation and calcification, we employed lentiviral flag-rictor overexpression during osteoblast differentiation. Rictor protein expression was increased 3.5 times after lentiviral transduction (Fig. 8A). Successful infection of MSC was also confirmed by detection of flag (Fig. 8A) and GFP (data not shown) that were also part of the plasmid. Similar to Rapa, overexpression of rictor resulted in mTORC2 activation as demonstrated by increased phosphorylation of AKT^Ser473^ (Fig. 8A). However, in contrast to Rapa-treated cells, mTORC1 signaling was not suppressed by rictor overexpression since levels of pp70S6K^Ser389^ were unchanged (Fig. 8A). In comparison to Rapa, rictor overexpression had similar effects on cell fates and phenotype: autophagic flux was stimulated (Fig. 8A), cellular senescence was prevented (Fig. 8A), anti-apoptotic signaling was increased (Fig. 8A) and cell death was reduced (Fig. 8B). Differentiation to calcifying osteoblast-like cells was inhibited as shown by reduced ALP activity (Fig. 8C) and less calcium deposition (Fig. 8D,E). These results suggest that protection from calcific transformation of MSC does not critically rely on mTORC1 inhibition but can be achieved with enhanced mTORC2 signaling alone.

### Rapa modulates mTOR signaling and activates protective cell fate patterns in vascular cells *in vivo*

**Figure 9:**
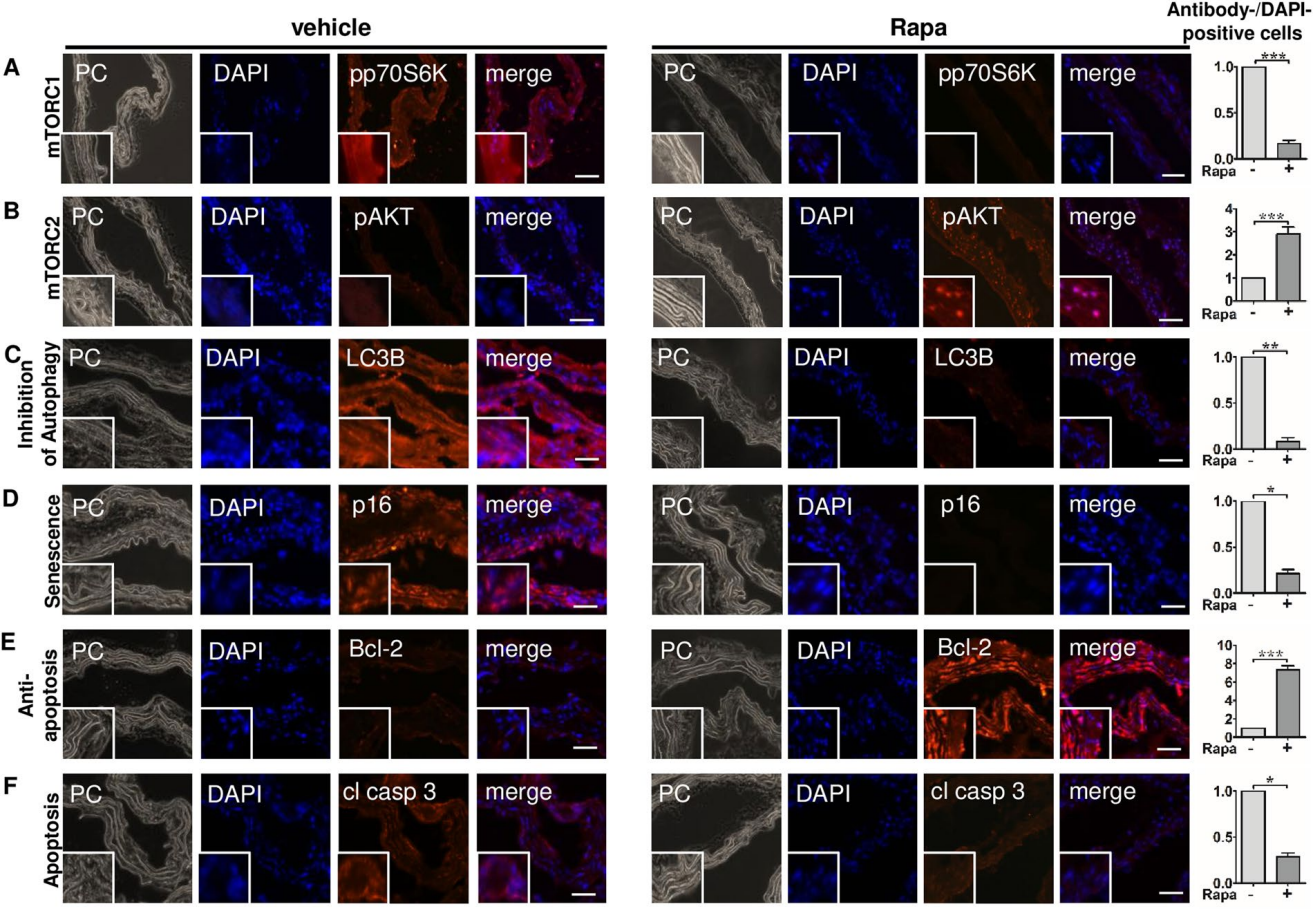
Rapa modulates mTOR signaling and activates protective cell fate patterns in vascular cells *in vivo*. Ten-week-old mice were exposed to Rapa (1.5 mg/kg) or vehicle for 37 days. 4 mice per group were studied. Sections from aortas were stained by immunofluorescence for (**A**) the mTORC1 downstream target pp70-S6KThr389, (**B**) the mTORC2 downstream target pAKTSer473, (**C**) LC3B with lower levels indicating increased degradation due to enhanced autophagic flux, (**D**) p16INK4a as a marker for cellular senescence, (**E**) Bcl-2, a negative regulator of apoptosis, (**F**) caspase 3, involved in apoptosis as an executioner caspase. Nuclei were counterstained with DAPI. Each panel shows a phase contrast micrograph (PC), DAPI staining alone (DAPI, blue fluorescence), the specific antibody staining alone (red fluorescence), and the merged image of a representative animal. Original magnification × 400, inserts 5× zoom, scale bar = 100 µm. The graphs show the proportion of antibody-positive cells out of 100 counted cells identified by DAPI stained nuclei. The vehicle group was set to 1. Bars represent mean + SEM, n = 4, *P < 0.05, **P < 0.01, ***P < 0.001.

As a proof-of-concept, we injected mice with vehicle or low doses of Rapa (1.5 mg/kg) for 37 days and examined local activation levels of mTORC1 and mTORC2 in cells of the aortic wall. By immunofluorescence, we found reduced levels of pp70S6K (Fig. 9A) and increased phosphorylation of AKT at Ser473 (Fig. 9B) in Rapa-treated animals compared to vehicle-treated controls. pAKT^Ser473^ was detected predominantly in the nucleus where it most potently counteracts apoptosis. LC3B II was suppressed showing increased autophagic flux (Fig. 9C). Expression of p16^INK4a^ was hardly detectable with Rapa indicating prevention of cellular senescence (Fig. 9D). Rapa strongly upregulated anti-apoptotic Bcl-2 (Fig. 9E) and blocked formation of cleaved caspase 3 (Fig. 9F) as a sign of suppressed apoptosis. Thus, Rapa induced the same mTOR signaling pattern and protective cell fate profile in murine vascular cells *in vivo* as observed in the cell culture model of calcifying human MSC.

## Discussion

We identified the major integrator of nutrient status and growth factor signaling, mTOR, as a decisive factor governing differentiation of human MSC to cells with osteoblast-like functionality. The central role of both mTOR complexes, mTORC1 and mTORC2, in the regulation of cell fate programs accounted for their fundamental influence on MSC osteoblastic differentiation. Activation of mTORC1 and mTORC2 was observed during osteoblastic differentiation and progressive calcification of MSC. This phenotype conversion was linked to blockade of autophagy, induction of cellular senescence, and initiation of apoptotic cell death. Pharmacologic intervention with Rapa potently prevented osteoblast differentiation and calcification by inhibition of mTORC1 and reciprocal activation of mTORC2 associated with alternative cell fate programs: early stimulation of autophagy was evident before phenotypic changes occurred followed by reduced cellular senescence and apoptosis. Mechanistically, preservation of an undifferentiated state critically depended on enhanced mTORC2 signaling via AKT and maintenance of autophagic flux as revealed by pharmacologic and genetic studies. *In vivo* experiments in mice confirmed that protective mTOR signaling and cell fate patterns antagonizing osteoblastic differentiation and calcification *in vitro* can be induced in artery walls by systemic administration of Rapa. We provide a rationale for therapeutic mTOR modulation to prevent exhaustion of the regenerating MSC pool and to protect from vascular calcification due to age and metabolic diseases. Furthermore, mTOR can be targeted to enhance osteoblastic differentiation of MSC in cell therapeutic approaches for degenerative bone diseases and osseous defects.

### Degeneration and regeneration depend on cell fate patterns controlled by mTOR

Loss of regenerative capacity to maintain the functional reserve of vital organs is a physiologic, age-related phenomenon leading to impaired stress resistance^16^. Individual internal and external risk factors such as genetic background, chronic metabolic conditions, and environmental circumstances as well as acute insults can accelerate this process^16,42,43^ and increase risk for diseases and premature death. Modulation of mTOR signaling has been shown to increase lifespan both on the single cell and organism level in yeast^44^, helminths^45^, flies^46^ and mammals^29,47^. As a potential mechanism, interference with cell fates controlled by mTOR in response to stress and metabolic cues has been discussed^48^. Autophagy, regulated chiefly by mTORC1, is accorded a central role in the preservation of juvenile cell adaptability^48,49^ since it exercises a double function as a survival mechanism in cellular stress conditions: during starvation, when mTORC1 is physiologically inhibited, autophagy regenerates basal metabolic precursors by “self-cannibalism” of cellular structures^24^. On the other hand, cellular debris such as misfolded proteins and dysfunctional organelles that can induce senescence and apoptosis is cleared by autophagy^50^. In our cell culture model of osteoblastic differentiation of MSC, reduced autophagy was the first detectable cell fate change in response to calcifying conditions. Modulation of mTOR signaling with Rapa potently maintained autophagic flux as indicated by lower levels of LC3B II and p62 due to lysosomal degradation and effectively ameliorated calcification. Conversely, blockade of autophagy with continuous, low-dose administration of bafilomycin A1 resulting in accumulation of autophagosomal LC3B II and p62, demonstrating reduced autophagic flux precipitated osteoblastic differentiation and calcium deposition. This argues that autophagy can be ascribed a central position in the transition from undifferentiated MSC to osteoblast-like calcifying cells.

Cellular senescence and apoptotic cell death followed reduced autophagy later in the time course of MSC differentiation to osteoblasts, suggesting that these cell fate changes might be secondary. However, Rapa indirectly activated mTORC2 whose downstream target AKT provides anti-apoptotic effects via inhibition of FOXO^28^. The importance of apoptosis for vascular calcification is supported by studies in VSMC demonstrating that apoptotic bodies from dying VSMC form a nidus to nucleate apatite^41^. Furthermore, apoptotic cells are specifically found in calcifying areas of arteries from patients with arteriosclerosis^51^. Thus, resistance to apoptosis by activation of survival mechanisms via mTORC2/AKT appears to be another important mechanism contributing to protection from calcific transformation of MSC besides enhanced autophagy.

It was reported that Rapa treatment preserved undifferentiated stem cell function and osteogenic differentiation potential during prolonged culture and expansion of MSC *in vitro* while senescence and DNA damage were reduced^52^. Interestingly both maintenance of fully functional MSC in their stem cell niche and resistance to calcifying stimuli rely on cellular functions that are associated with youth and longevity, progressively decrease during aging, and can be enhanced by mTOR modulation with Rapa. We propose that age-related arterial calcification and accelerated arteriosclerosis in chronic metabolic diseases share inappropriate function of vascular progenitors due to a preponderance of adverse cell fates over regenerative ones. Enabling protective cell fate patterns in the MSC-pericyte-VSMC-continuum could be a novel approach for prevention and treatment of vascular diseases.

### Harnessing the mTOR network for endogenous and exogenous regenerative approaches

The most striking finding of our study is that Rapa-mediated blockade of osteoblastic differentiation and calcification was not solely due to inhibition of mTORC1 but crucially depended on enhanced mTORC2 signaling. Rapa is an inhibitor of mTORC1 without direct effects on mTORC2. However, depending on cell type, tissue, and duration of exposure, Rapa can either indirectly activate or inhibit signaling via mTORC2^33,40^. During osteoblastic differentiation of MSC, Rapa was a potent activator of mTORC2 throughout the whole time course of three weeks. Genetic ablation of mTORC2 by targeting its obligatory constituent rictor with shRNA completely abolished the differentiation blockade induced by Rapa and pharmacologic inhibition of the mTORC2 downstream target AKT had the same effect. Conversely, isolated activation of mTORC2 by rictor overexpression without mTORC1 inhibition was sufficient to confer protection from calcification. Thus, mTORC2-dependent cell functions are essential and appear to be even more important for Rapa-mediated antagonism of differentiation than the counteraction of processes classically controlled by mTORC1 such as ribosomal biogenesis and protein synthesis. Our results suggest an as-yet-unknown role for mTORC2 in the preservation of pluripotency in stem and progenitor cells.

Activated mTORC1 signaling, as found in MSC in transition to calcifying osteoblasts, is the signature status of the mTOR network in conditions with prominent cardiovascular pathologies such as chronic nutrient overload, obesity, and diabetes mellitus type 2^21^. Inhibition of mTORC1 with reciprocal activation of mTORC2 has been reported to confer protection in cardiovascular diseases. When mTORC2 signaling was induced as a consequence of mTORC1 inhibition by either Rapa^37^ or overexpression of PRAS40^53,54^, hearts were protected from maladaptive hypertrophy and pathologic remodeling as well as from cardiomyocyte apoptosis. Rapa attenuated atherosclerosis in apolipoprotein E-deficient mice^55^ and vascular calcification in rats with chronic renal failure^56^. In a recent study, Rapa reduced aortic calcium load and prolonged survival in a mouse model for uremic vascular media calcification^57^. Notably, Rapa mediated protection was associated with induction of autophagy in aortic walls^57^. Our findings provide a mechanistic explanation for these observations, since we demonstrated that systemic administration of Rapa shifts mTOR signaling in favor of mTORC2 while suppressing mTORC1 in vascular cells *in vivo*. Similar to Rapa-treated MSC undergoing osteoblastic differentiation *in vitro*, autophagy was stimulated while senescence and apoptosis were reduced in vascular cells of Rapa-treated mice, implicating protection against arterial calcification through enhanced mTORC2 signaling.

Nevertheless, therapeutic interventions in patients aimed at reducing mTORC1 activity and increasing mTORC2 activity are hampered by several considerations. Rapa and similar mTORC1 inhibitors already approved for clinical use can also block instead of activate mTORC2 in some cells and settings with detrimental effects. As an example, transplanted patients receiving Rapa as an immunosuppressant can develop focal segmental glomerulosclerosis due to podocyte injury associated with reduced mTORC2 function^58^. In addition, the reciprocal activation of mTORC2 upon mTORC1 blockade by Rapa might be different in women and men since mouse hearts challenged by mineralocorticoid excess showed a sexual dimorphism in this regard^37^ and the positive effect of Rapa on lifespan in mice was greater in females than in males^47^. However, we included MSC from women and men in our study and did not observe any significant interaction between sex and the response to Rapa. Finally, activation of mTORC2 may have adverse effects such as facilitation of tumor growth due to reduced susceptibility to physiologic apoptotic stimuli. Alternatively, exogenous regenerative strategies might be advantageous because they offer the opportunity for selective intervention in a specific cell type *ex vivo*.

Autologous and allogenic MSC hold great potential for cell therapeutic treatment of hard-to-heal osseous lesions either due to developmental defects in pediatric patients or to osteoporotic fractures in the elderly. In accordance to our study, a biphasic approach could yield optimal results: During culture and expansion of MSC previous to implantation, activation of mTORC2 might preserve stemness and their functionality as progenitors, whereas activation of mTORC1 and simultaneous inhibition of mTORC2 in the differentiation phase might enhance osteoblastic output and improve calcification. During the expansion phase, selective activation of mTORC2 with novel pharmacologic or genetic methods seems advantageous over mTOR modulation with conventional mTORC1 inhibitors such as Rapa since mTORC1 blockade has profound anti-proliferative effects.

### Limitations

The use of bone marrow-derived cells to study vascular calcification could be questioned. Our aim was to specifically address progenitor cells to enhance endogenous regeneration as a more promising approach as compared to targeting terminally differentiated cells. Pericytes, the local progenitors to vascular smooth muscle cells, are very hard to isolate in sufficient numbers. Thus, we chose to study bone marrow-derived MSC that circulate and replenish pericytes in the vasculature^2,59,60^. This relationship and the reported similarities of pericytes and MSC^59^ give confidence that the same mechanisms are operative in both, pericytes located in the vessel wall and bone marrow derived MSC as their systemically circulating counterparts. Furthermore, MSC can easily be isolated from bone marrow aspirates, allowing for standardized experiments with MSC from multiple donors to account for inter-individual variation.

Although protein levels of the key subunits of mTORC1 and mTORC2, raptor and rictor, were not affected in osteoblast differentiation or by Rapa treatment, formation of one or both complexes might have changed. For example, recruitment of rictor to mTOR might be enhanced by Rapa resulting in increased formation of mTORC2 and phosphorylation of AKT at serine 473 despite unchanged protein levels. However, prolonged treatment with Rapa has been reported to inhibit formation of mTORC2 under certain conditions^33^. Investigations of protein-protein-interactions were beyond the scope of the present study.

Bafilomycin A1 blocks autophagy by inhibition of the V-ATPase preventing acidification of lysosomes and by blockade of autophagosome-lysosome fusion. Furthermore, bafilomycin A1 can act as a potassium ionophore^61^. Although various cell types may have different sensitivities to ionophoric activities and bafilomycin A1 has a rather low affinity for potassium ions^62^ we cannot verify that the effects of bafilomycin A1 in our study were solely related to inhibition of autophagy and not to its ionophoric action.

We did not validate our findings in an *in vivo* model for vascular calcification. However, Rapa induced exactly the same signaling patterns and effects on cell fates in vascular cells *in vivo* as we observed in our *in vitro* model. These findings confirm the concept of induction of regenerative cell fate patterns in vascular cells by activation of mTORC2. This novel therapeutic intervention could be beneficial in several vascular pathologies beyond vascular calcification and should therefore be tested in various *in vivo* models of vascular damage in the future.

## Conclusion

The bone-vascular axis is controlled by endocrine and metabolic signals impinging on the mTOR network and is progressively dysfunctional with aging and in chronic diseases, leading to loss of bone mass while vascular calcification progresses. MSC, capable of osteoblastic differentiation and calcification, have a dual role as progenitors to osteoblasts and as pericytes to VSMC. We found that degenerative cell fates associated with both, aging and metabolic diseases such as cellular senescence and apoptosis were activated during osteoblast differentiation of MSC while autophagy as a protective mechanism was reduced. Concomitant treatment with Rapa “rejuvenated” MSC by inducing autophagy and reducing cellular senescence and apoptosis via inhibition of mTORC1 and simultaneous activation of mTORC2 resulting in potent reduction of osteoblastic differentiation and calcification. We propose that therapeutic interventions aimed at the preservation of the regenerative capacity of vascular progenitor cells is most promising for the prevention of chronically progressing conditions such as arteriosclerosis as well as for the treatment of already established vascular lesions by enabling endogenous regeneration. Our study identified mTORC2, a thus far scarcely studied part of the mTOR network, as a powerful molecular target for maintenance of an undifferentiated state in MSC. Targeted interventions, particularly in the elderly and in patients with chronic metabolic diseases, may help restore a functional bone-vascular axis to reduce ectopic osteoblastic differentiation of MSC leading to prevention of osteoporotic fractures and improved vascular health.
