# Publisher Correction: mTORC1 and mTORC2 Differentially Regulate Cell Fate Programs to Coordinate Osteoblastic Differentiation in Mesenchymal Stromal Cells

**DOI:** 10.1038/s41598-020-59865-9

**Published:** 2020-02-25

**Authors:** Theres Schaub, Dennis Gürgen, Deborah Maus, Claudia Lange, Victor Tarabykin, Duska Dragun, Björn Hegner

**Affiliations:** 1 Clinic for Nephrology and Intensive Care Medicine, Charité-Universitätsmedizin Berlin, corporate member of Freie Universität Berlin, Humboldt-Universität zu Berlin, and Berlin Institute of Health, Berlin, Germany; 2 Institute for Chemistry and Biochemistry, Freie Universität Berlin, Berlin, Germany; 3 Institute of Cell Biology and Neurobiology, Charité-Universitätsmedizin Berlin, corporate member of Freie Universität Berlin, Humboldt-Universität zu Berlin, and Berlin Institute of Health, Berlin, Germany; 4 Center for Cardiovascular Research (CCR), Charité University Hospital, Berlin, Germany; 5 Experimental Pharmacology & Oncology Berlin-Buch GmbH, Berlin, Germany; 6 Junior Research Group 2: Metabolism of Microbial Pathogens, Robert Koch Institute, Berlin, Germany; 7 Clinic for Stem Cell Transplantation, Department of Cell and Gene Therapy, University Medical Center Hamburg-Eppendorf, Hamburg, Germany; 8 Berlin-Brandenburg School for Regenerative Therapies (BSRT), Berlin, Germany; 9 Vivantes Ida Wolff Hospital for Geriatric Medicine, Berlin, Germany

Correction to: *Scientific Reports* 10.1038/s41598-019-56237-w, published online 27 December 2019

In the original version of this Article, Björn Hegner was incorrectly listed as the corresponding author. The correct corresponding author for this Article is Duska Dragun. Correspondence and request for materials should be addressed to duska.dragun@charite.de. This error has now been corrected in the HTML and PDF versions of the Article.

